# Association of Lifestyle and Food Consumption with Bone Mineral Density among People Aged 50 Years and Above Attending the Hospitals of Kathmandu, Nepal

**DOI:** 10.1155/2019/1536394

**Published:** 2019-05-22

**Authors:** Narendra Kumar Chaudhary, Mukti Nath Timilsena, Dev Ram Sunuwar, Pranil Man Singh Pradhan, Raj Kumar Sangroula

**Affiliations:** ^1^Department of Radiology, Nepal Orthopaedic Hospital, Kathmandu, Nepal; ^2^Department of Orthopaedic Surgery, Nepal Orthopaedic Hospital, Kathmandu, Nepal; ^3^Department of Nutrition and Dietetics, Armed Police Force Hospital, Kathmandu, Nepal; ^4^Department of Community Medicine and Public Health, Institute of Medicine, Tribhuvan University, Nepal; ^5^Department of Nutrition and Dietetics, College of Applied Food and Dairy Technology, Kathmandu, Purbanchal University, Nepal

## Abstract

**Background:**

Bone mineral density (BMD) is the measure of the minerals, mostly calcium and phosphorous, contained in certain volume of bone to diagnose osteoporosis. The aim of the study was to find out the association of lifestyle and food consumption with BMD.

**Methods:**

An analytical cross-sectional study was conducted among 169 people of age 50 years and above who underwent Dual Energy X-Ray Absorptiometry (DEXA or DXA) scan in the hospitals of Kathmandu valley of Nepal. Food frequency questionnaire and 24-hour recall methods were followed. The DXA reports of the participants were observed to identify osteoporosis. Chi-square test, independent sample t-test, and binary logistic regression were applied to explore the association of BMD with different variables.

**Result:**

The prevalence of osteoporosis, osteopenia, and normal BMD was 37.3%, 38.5%, and 24.2%, respectively. The prevalence of osteoporosis increased with sex and age (AOR 3.339, CI: 1.240-8.995, p-value 0.017; AOR 3.756, CI: 1.745-8.085, p-value 0.001), respectively. Higher BMI was associated with lower odds of osteoporosis (AOR 0.428, CI: 0.209-0.877, p-value 0.020). Smoking had bad effect on the health of bone (AOR 3.848, CI: 1.179-12.558, p-value 0.026). Daily dietary calcium intake had negative association with osteoporosis with the p-value of 0.003; however, the daily consumption of vitamin D rich food had no association with osteoporosis.

**Conclusion:**

High prevalence of osteoporosis and osteopenia was found in older people. Osteoporosis was found to be significantly associated with sex, age, lower BMI, smoking habit, and daily calcium consumption. Further research can be conducted by making the relationship of calcium consumption with the numerical T-value of scanned body parts.

## 1. Introduction

Bone density usually refers to the degree to which a radiation beam is attenuated by a bone, as judged from a two-dimensional projection image (areal bone density) in clinical practice and science [1]. The purpose of bone densitometry is to diagnose the osteoporosis, to evaluate fracture risk, and to determine whether treatment is required or not. There is a statistical association between poor bone density and higher probability of fracture [2]. Poor bone mass leads to the fracture with low energy trauma [3].

Osteoporosis is a progressive skeletal disease. It is characterized by low bone mass and microarchitectural deterioration of bone tissue structure, with a consequent increase in bone fragility and susceptibility to fractures [4]. It is a silent disease that appears later in life. The prevalence of osteoporosis is very high worldwide. One in two women and one in five men aged 50 years or over will suffer an osteoporotic fracture [5]. About 10% of US adults of age 50 years and older had osteoporosis and 43.9% had low bone mass at the femoral neck or lumbar spine in NHANES 2005–2010 census study [6]. The prevalence of osteoporosis was 50% and that of osteopenia was 36% in the persons above 50 years old in the hospitalized setting, a tertiary care centre of South India [7]. It has been found that 90% of the postmenopausal women had subnormal T-scores in the Moradabad of India [8]. Very few studies about osteoporosis are found in the context of Nepal. The prevalence of osteoporosis among the middle aged women in Chitwan district of Nepal is 26.2%, osteopenia is 39.3%, and normal BMD is 34.4% at wrist site [9]. Another study indicates that 15% of postmenopausal women had osteoporosis and 21% had osteopenia in the Gandaki Medical College Teaching Hospital, Pokhara of Nepal [10].

Osteoporosis may cause the patients to be bedridden with back pain, loss of height, kyphosis, pneumonia, and pulmonary thromboembolism. The treatment costs are the most expensive after diabetes, hyperlipidaemia, hypertension, and heart diseases [11]. Lifestyle and dietary behavior are important factors for the health of bone [12]. Deficiency of calcium and vitamin D contributes to alterations of bone remodeling and bone integrity [13]. Dietary calcium has significant positive association with higher BMD at all sites of our body [14].

This study has attempted to explore the association of lifestyle and food consumption with BMD among people of age 50 years and above in Kathmandu of Nepal by analyzing the DXA report. DXA is considered as the gold standard method for bone densitometry. The precision error of current DXA systems is 1.0% for whole body, ~0.5-1% for the spine, and 2.0-5.0% for the femoral neck, depending on anatomic site analyzed [15]. This technology is recently brought in Nepal and available in very few hospitals of Kathmandu. Therefore there is a lack of studies about BMD through DXA report. Besides these, the cost of DXA scan is high, so this investigation is not normally preferred for patients with low economic background. That is why there is no authentic data for the prevalence of osteoporosis in Nepal as well.

## 2. Materials and Methods

The study was a cross-sectional analytical study conducted over a duration of six months from October 2017 to March 2018. This study was registered and approved by the Research and Institutional Review Committee (IRC) of Nepal Medical College Teaching Hospital (NMCTH) (Reference number: 33-074/075). The respondents of the study were the patients of age 50 years and above who had undergone Dual Energy X-Ray Absorptiometry (DXA). Written informed consent was taken from the participants. The study was conducted at the four super-speciality hospitals of Kathmandu where DXA facility was available. As this study was hospital based, we used the consecutive sampling method until the sample size was met. The patients who were seriously ill, fractured case, and having DXA report of sites other than lumbosacral spine and both femurs were excluded from the study.

The sample size was determined by using a single proportional formula using the prevalence rate of osteoporosis in India. The prevalence of osteoporosis in patients aged 50 years and above was 50% according to studies from South India [7]. We determined sample size by taking 95% confidence interval (CI) and 8% margin of error (d). Hence, with 13% nonresponse rate, the total sample size would be 169. Data collection was done through the observation of DXA reports of patients. All the hospitals under study had the same manufacturer machine, GE Lunar Prodigy. Before conducting the DXA scan, the machine was calibrated by the concerned operators for the accuracy of the diagnosis. Food habit and lifestyle factors were explored through 24-hour dietary recall and food frequency questionnaire survey. For this, a questionnaire was developed and pilot study was done.

Data were entered into EpiData version 3.2 and analyzed by using SPSS 20. The prevalence of osteoporosis in older people was estimated through descriptive statistical tools using WHO criteria for osteoporosis (normal BMD if T-score is equal to or above -1 S.D., osteopenia if T-score ranges between -1 S.D. and -2.5 S.D, and osteoporosis if T-score is equal to or below -2.5 S.D). Subjects were classified as having osteoporosis if at least one of the two measurements (lumbar spine or femur) indicated osteoporosis and as having osteopenia if at least one measurement indicated osteopenia, but none indicated osteoporosis. The pattern of consumption of calcium rich food was explored qualitatively. Sufficient intake was estimated through food habit pattern. The categorical independent lifestyle related variables such as smoking, exercise, and alcohol consumption were explored. Through the 24-hour recall, dietary daily calcium intake was calculated manually with the help of food composition table 2012 of Nepal [16]. Similarly daily dietary vitamin D consumption was calculated by using food composition table 2017 of India [17]. Measurement list for the nutritive value (calcium and vitamin D) per household was prepared for the ease of calculation. The household measurement of simple and traditional tea glass of 130 ml was displayed while interviewing respondents for approximate value, as per the principles and guidelines made by the Dietary Department of Tribhuvan University Teaching Hospital (TUTH), Kathmandu, Nepal, and also the guidelines of ASTHA Nepal. Chi-square test and independent t-test were applied to find out the association of different variables with BMD. BMD was further categorized as osteoporosis and no osteoporosis. No osteoporosis was considered both for osteopenia and normal BMD. All associations with probability values less than 0.05 (p < 0.05) have been considered statistically significant. Binary logistic regression analysis was used for the multivariate analysis of p-value less than 0.05 (p<0.05).

## 3. Results

The aim of the study was to find out the association of lifestyle and food consumption with bone mineral density among people of age 50 years and above attending the hospitals of Kathmandu. Most of the patients were 60 years old and above and females (Table 1).

Most of the patients were nonvegetarian. About 90% of the participants had less calcium intake than DRI (Table 2).

The prevalence of osteoporosis, osteopenia, and normal BMD was measured both at lumbosacral spine and femoral neck. Overall 24.2% had normal BMD, 38.5% had osteopenia, and 37.3% had osteoporosis (Table 3).

The incidence of osteoporosis was more in females than in males. Similarly osteoporosis was positively associated with age and smoking while it was negatively associated with BMI; however it was not associated with daily exercise and daily tea consumption (Table 4).

The daily calcium intake had good effect on the health of bone while daily dietary vitamin D intake had no association with osteoporosis (Table 5).

## 4. Discussion

### 4.1. Prevalence of Osteoporosis

Prevalence of osteoporosis increased with age and it was more in females [18, 19]. Prevalence of the osteoporosis was found to be 37.3% in people of age 50 years and above, which is less than that in the findings of the study done in India [7]. It may be because of application of quantitative ultrasound (QUS) in those studies. QUS screening for osteoporosis is appropriate for younger perimenopausal woman due to its mode of action and, hence, not appropriate for older people as the bone of older patients is too compact to allow penetration by ultrasound [18]. Different cut-off values for diagnosis are used by different QUS devices that underestimate the true prevalence of osteoporosis, which indicates the poorer precision compared to DXA [19]. Another study also indicated higher prevalence of osteoporosis among the postmenopausal women from QUS than DXA methods [20]. The prevalence of osteoporosis was 9% in females of age group 50-60 years while there was no osteoporosis in males of the same age group in Pokhara by Peripheral Radial Densitometry [10]. Similarly, the prevalence of osteoporosis was 26.2% in premenopausal and postmenopausal women in Chitwan district of Nepal through the quantitative ultrasound technology at wrist joints [9]. However, peripheral skeletal sites are not clinically useful to monitor the changes in BMD for the diagnosis of osteoporosis [21].

### 4.2. Lifestyle and BMD

There are many lifestyle factors that affect BMD. Participants with normal BMI had high prevalence of occurring osteoporosis than people who were overweight or obese. The higher body weight makes a huge mechanical load on the bone, hence increasing the bone mass to accommodate this load, and for this the body fat acts to exert a protective factor for fracture [22]. However it is important to remember that not all types of fat are beneficial for bone mass. Low BMI individuals lose more bone compared to those with higher BMI individuals [23]. Similarly, the smokers had three times more probability of having osteoporosis than nonsmokers which is supported by different studies [24, 25]. Smoking decreases the calcium absorption [26]. Hence it decreases the bone mineral density and increases the risk of sustaining the fractures or tendon injury [27]. Alcohol consumption had protective effect on bone health from bivariate analysis. Participants who consumed moderate level of alcohol, that is, 0.5 to 1 drinks (15 ml to 30 ml) per day, had a lower risk of hip fracture [28]. However, high consumption of alcohol increases the calcium lost in urine which reduces the bone mass [29].

Several studies revealed that osteoporosis was not associated with daily exercise [30, 31] which was also found in this study. Daily tea consumption did not show any beneficial effect on the improvement of BMD. However, consumption of tea has good effect on bone mineral density [32, 33]. This may be due to the difference in frequency and concentration of consumption of tea. Studies have shown that mean consumption of milk tea was five glasses with coffee per day while in the present study most of the participants consumed two glasses of black tea. The caffeine concentration in tea is generally less than 50% of the concentration found in coffee [34], and the bioactive components of tea support the osteoblastic activities and suppress the osteoclastic activities [35].

### 4.3. BMD with Calcium Consumption and Vitamin D Intake

The mean intake of calcium consumption was found to be 520 mg which is similar to the study done in South India [36]. It was found that osteoporosis was negatively associated with daily calcium intake which has been supported by different studies [14, 37, 38]. There was no association of vitamin D intake from food with osteoporosis. This finding has been supported by another study [39]. This type of finding may be due to the fact that 50-90% of total vitamin D requirement can be fulfilled by sun exposure, and only 5-10% of total vitamin D can be achieved from food [11].

This study has several limitations. Sample size was less due to unavailability of patients as DXA facility is a new and expensive technology in the context of Kathmandu, Nepal. Similarly, the food composition table 2012 of Nepal does not include calcium value of many foods like momo (a type of South Asian dumpling) which is mostly consumed in Kathmandu.

## 5. Conclusion

About three-fourths of people of age 50 years and above had lower BMD suffering from the osteopenia and osteoporosis. The high prevalence of osteopenia and osteoporosis is positively associated with the daily low calcium intake from foods, lower BMI, smoking habit, sex especially female, and increasing age after 50 years. Therefore older people should be prioritized for calcium rich food and supplementation for the prevention of osteoporosis. As this study could not make any relationship of the numerical T-score value with the amount of calcium intake, further study can be conducted from this perspective.

## Figures and Tables

**Table 1:** Characteristics of respondents (n=169).

Variables	Mean± S.D.

Age (in years)	63.46± 9.784
Height	152.5760±7.32034
Weight	60.0994±11.59607
BMI	25.7784±4.48414
Daily calcium intake (in mg)	520.4488±296.97648
Daily dietary vitamin D intake (in IU)	578.6688±435.53989
BMD value (T-score in S.D.)	
LS Spine-AP	-1.6976±1.66812
Right Femur-AP	-1.2284±1.38104
Left Femur-AP	-1.2538±1.37720

Variables	Frequency (%)

*Sex*	
Male	38(22.5%)
Female	131(77.5%)
*Age*	
50 to 59 Years	67(39.6%)
60 years and above	102(60.4%)
*BMI*	
Underweight	0(0%)
Normal	71(42%)
Overweight	98(58%)
*Smoking habit*	
Yes	21(12.4%)
No	148(87.6%)
*Alcohol intake*	
Yes	53(31.4%)
No	116(68.6%)
*Daily Tea intake*	
Yes	149(88.2%)
No	20(11.8%)
*Daily exercise*	
Yes	67(39.6%)
No	102(60.4%)

**Table 2:** Dietary habit of respondents (n=169).

Variables	Frequency (%)
*Food Habit*	
Vegetarian	22(13%)
Non-vegetarian	147(87%)
*Milk and milk products consumption*	
Daily	109(64.5%)
Once a week	60(35.5%)
*Green Vegetables consumption*	
Daily	136(80.5%)
Once in week	33(19.5%)
*Fish consumption*	
Never	22(13%)
Daily	20(11.8%)
Once in week	127(75.1%)
*Daily calcium intake*	
<500 mg	92(54.44%)
500 to 999 mg	71(42.01%)
≥1000 mg	6(3.55%)

**Table 3:** Prevalence of osteoporosis (n=169).

Respondents	Normal	Osteopenia	Osteoporosis	Total
*Age*				
50 to 59 years	23(34.3%)	31(46.3%)	13(19.4%)	67(39.6%)
60 years and above	18(17.6%)	34(33.33%)	50(49.1%)	102(60.4%)
*Sex*				
Male	12(31.6%)	17(44.7%)	9(23.7%)	38(22.5%)
Female	29(22.1%)	48(36.6%)	54(41.2%)	131(77.5%)
*Total*	41(24.2%)	65(38.5%)	63(37.3%)	169(100%)

**Table 4:** Association of different variables with BMD.

Variables	No osteoporosis	Osteoporosis	Bivariate analysis		Multivariate analysis	
			p-value	COR(95%CI)	p-value	AOR(95%CI)
*Sex*						
Male	29	9		Ref		Ref
Female	77	54	0.049*∗*	2.260(0.990-5.516)	0.017*∗∗*	3.339(1.240-8.995)
*Age*						
50-59 years	54	13		Ref		Ref
60 years and above	52	50	<0.001*∗*	3.994(1.946-8.200)	0.001*∗∗*	3.756 (1.745-8.085)
*BMI*						
Normal	34	37	0.001*∗*	3(1.6-5.7)	0.020*∗∗*	2.339(1.141-4.795)
Overweight/obese	72	26		Ref		Ref
*Smoking*						
Yes	9	12	0.04*∗*	2.534(1.002-6.417)	0.026*∗∗*	3.848(1.179-12.558)
No	97	51		Ref		Ref
*Alcohol consumption*						
Yes	40	13	0.021*∗*	0.429(0.208-0.886)	0.143	0.525 (0.221-1.244)
No	66	50		Ref		Ref
*Daily exercise*						
Yes	48	19	0.052	0.522(0.270-1.010)	—	—
No	58	44		Ref		
*Tea consumption*						
Yes	95	54	0.447	0.659(0.271-1.782)		
No	11	9		Ref	—	—

*∗* denotes significant variables, P-value < 0.05, for bivariate analysis.

*∗∗* denotes significant variables, P-value < 0.05, for multivariate analysis.

COR: crude odds ratio.

AOR: adjusted odds ratio.

Ref: reference category.

**Table 5:** Dietary association with BMD.

Result	Frequency	Mean± SD	P-Value
*Calcium consumption (in mg)*			
No osteoporosis	106	572.32**±**332.52	0.003*∗*
Osteoporosis	63	433.17**±**198.43	
*Vitamin D intake (in IU)*		574.19**±**314.82	
No osteoporosis	106	586.19**±**588.19	0.863
Osteoporosis	63		

*∗* denotes significant variable, P-Value<0.05, for independent sample t-test.

## Data Availability

The data used to support the findings of this study are available from the corresponding author upon request.

## Supplementary Materials

Supplementary 1 S1 Dataset.

Supplementary 2 S2 STROBE checklist.
