# Supplementary material for: Association of Lifestyle and Food Consumption with Bone Mineral Density among People Aged 50 Years and Above Attending the Hospitals of Kathmandu, Nepal
Source: J Osteoporos. 2019 May 22;2019:1536394. doi: 10.1155/2019/1536394 (PMC6556264; doi:10.1155/2019/1536394)
Supplement: Supplementary 2 — S2 STROBE checklist. [file 1536394.f2.pdf]

STROBE Statement—checklist of items that should be included in reports of observational studies

	Item No.	Recommendation	Page No.	Relevant text from manuscript
**Title and abstract**	1	(*a*) Indicate the study's design with a commonly used term in the title or the abstract	1	"Lifestyle" "food consumption" "bone mineral density"
		(*b*) Provide in the abstract an informative and balanced summary of what was done and what was found	2	Cross-sectional study was conducted among 169 people using Dual Energy X-ray Absorptiometry (DEXA or DXA) scan. High prevalence of osteoporosis among old age people.
**Introduction**				
Background/rationale	2	Explain the scientific background and rationale for the investigation being reported	2 & 3	
Objectives	3	State specific objectives, including any prespecified hypotheses	3	To explore the association of lifestyle and food consumption with BMD among the people of age 50 years and above in Kathmandu of Nepal by analysing the DXA report.
**Methods**				
Study design	4	Present key elements of study design early in the paper	3 & 4	Analytical cross-sectional study.
Setting	5	Describe the setting, locations, and relevant dates, including periods of	4 & 5	The study was conducted

		recruitment, exposure, follow-up, and data collection		at the four super-speciality hospitals of Kathmandu where DXA facility was available. Study was conducted over 6 months from October 2017 to March 2018.
Participants	6	(*b*) *Cohort study*—For matched studies, give matching criteria and number of exposed and unexposed<br>*Case-control study*—For matched studies, give matching criteria and the number of controls per case<br>*Cross-sectional study*—Give the eligibility criteria, and the sources and methods of selection of participants		
		Cross-sectional study—Give the eligibility criteria, and the sources and methods of selection of participants	3	The inclusion criteria of this study was those participants aged 50 years or above attending DXA scan in the radiology department of hospitals and we excluded participants who were seriously ill, sustained fracture case and DXA report sites of other than lumbosacral spine and both femurs
Variables	7	Clearly define all outcomes, exposures, predictors, potential confounders, and effect modifiers. Give diagnostic criteria, if applicable	4	**Dependent Variables:**<br>Osteoporosis (T score ≤ -2.5 S.D.) and no

				osteoporosis<br>**Independent Variables**<br>Lifestyle: smoking, exercise and alcohol consumption.<br>**Dietary consumption**<br>Daily Calcium intake from food, Vitamin D intake through food consumption, Tea and coffee consumption
Data sources/ measurement	8*	For each variable of interest, give sources of data and details of methods of assessment (measurement). Describe comparability of assessment methods if there is more than one group		
Bias	9	Describe any efforts to address potential sources of bias		
Study size	10	Explain how the study size was arrived at	4	The sample size was calculated based on the similar study conducted in India and we took prevalence of osteoporosis in patient 50 years and above was 50% from these studies.

Continued on next page

Quantitative variables	11	Explain how quantitative variables were handled in the analyses. If applicable, describe which groupings were chosen and why	4 & 5	Data were entered in Epi Data and exported to SPSS

				20.0 for statistical analysis.
Statistical methods	12	(*a*) Describe all statistical methods, including those used to control for confounding		Descriptive analysis was used to socio-demographic variables. Chi-square test was used to apply for bivariate analysis and multivariate binary logistic regression was applied to adjusting possible confounders. One sample t-test was used for continuous variable.
		(*b*) Describe any methods used to examine subgroups and interactions		Binary logistic regression was used
		(*c*) Explain how missing data were addressed		Before analysis, all data were re-check thoroughly and cleaning.
		(*d*) *Cohort study*—If applicable, explain how loss to follow-up was addressed<br>*Case-control study*—If applicable, explain how matching of cases and controls was addressed<br>*Cross-sectional study*—If applicable, describe analytical methods taking account of sampling strategy		
		(*e*) Describe any sensitivity analyses		
**Results**				
Participants	13*	(a) Report numbers of individuals at each stage of study—eg numbers potentially eligible, examined for eligibility, confirmed eligible, included in the study, completing follow-up, and analysed		
		(b) Give reasons for non-participation at each stage		
		(c) Consider use of a flow diagram		
Descriptive data	14*	(a) Give characteristics of study participants (eg demographic, clinical, social) and information on exposures and potential confounders	5 & 6	Characteristics of participants is presented in

				table 1.
		(b) Indicate number of participants with missing data for each variable of interest		
		(c) *Cohort study*—Summarise follow-up time (eg, average and total amount)		
Outcome data	15*	*Cohort study*—Report numbers of outcome events or summary measures over time		
		Case-control study—Report numbers in each exposure category, or summary measures of exposure		
		Cross-sectional study—Report numbers of outcome events or summary measures	6 & 7	All relevant report is present in table 2 & 3.
Main results	16	(*a*) Give unadjusted estimates and, if applicable, confounder-adjusted estimates and their precision (eg, 95% confidence interval). Make clear which confounders were adjusted for and why they were included	8	Bivariate and multivariate logistic regression report is presented in table 4.
		(*b*) Report category boundaries when continuous variables were categorized		
		(*c*) If relevant, consider translating estimates of relative risk into absolute risk for a meaningful time period		

Continued on next page

Other analyses	17	Report other analyses done—eg analyses of subgroups and interactions, and sensitivity analyses		
**Discussion**				
Key results	18	Summarise key results with reference to study objectives	9, 10, & 11	
Limitations	19	Discuss limitations of the study, taking into account sources of potential bias or imprecision. Discuss both direction and magnitude of any potential bias	11	
Interpretation	20	Give a cautious overall interpretation of results considering objectives, limitations, multiplicity of analyses, results from similar studies, and other relevant evidence		
Generalisability	21	Discuss the generalisability (external validity) of the study results		
**Other information**				
Funding	22	Give the source of funding and the role of the funders for the present study and, if applicable, for the original study on which the present article is based	12	Author declare that they have no conflict of interest exist.

*Give information separately for cases and controls in case-control studies and, if applicable, for exposed and unexposed groups in cohort and cross-sectional studies.

Note: An Explanation and Elaboration article discusses each checklist item and gives methodological background and published examples of transparent reporting. The STROBE checklist is best used in conjunction with this article (freely available on the Web sites of PLoS Medicine at http://www.plosmedicine.org/, Annals of Internal Medicine at http://www.annals.org/, and Epidemiology at http://www.epidem.com/). Information on the STROBE Initiative is available at www.strobe-statement.org.
